# Design and Practical Stability of a New Class of Impulsive Fractional-Like Neural Networks

**DOI:** 10.3390/e22030337

**Published:** 2020-03-15

**Authors:** Gani Stamov, Ivanka Stamova, Anatoliy Martynyuk, Trayan Stamov

**Affiliations:** 1 Department of Mathematics, Technical University of Sofia, 8800 Sliven, Bulgaria; gstamov@abv.bg; 2 Department of Mathematics, University of Texas at San Antonio, San Antonio, TX 78249, USA; 3 S.P. Timoshenko Institute of Mechanics, NAS of Ukraine, 03057 Kiev-57, Ukraine; center@inmech.kiev.ua; 4 Department of Machine Elements and Non-metallic Constructions, Technical University of Sofia, Sofia 1000, Bulgaria; tstamov@tu-sofia.bg

**Keywords:** neural networks, fractional-like derivative, impulses, practical stability, *h*−manifolds

## Abstract

In this paper, a new class of impulsive neural networks with fractional-like derivatives is defined, and the practical stability properties of the solutions are investigated. The stability analysis exploits a new type of Lyapunov-like functions and their derivatives. Furthermore, the obtained results are applied to a bidirectional associative memory (BAM) neural network model with fractional-like derivatives. Some new results for the introduced neural network models with uncertain values of the parameters are also obtained.

## 1. Introduction

Cellular neural network systems [1,2] and their various generalizations have attracted the attention of the researchers due to their incredible opportunities for applications in areas such as pattern recognition, associative memory, classification, parallel computation, as well as, their ability to solve complex optimization problems. As such, neural network models have promising potential for applications in numerous engineering tasks [3,4], including engineering design tasks [5]. It is also observed that an efficient neural network’s training is related to entropy phenomena [6]. In addition, in many cases, entropy is used to measure the complexity in a neural network architecture [6,7].

On the other hand, since impulsive phenomena may affect the neural network behavior, some important and interesting results about different classes of impulsive neural networks have been obtained. See, for example [8,9,10,11,12,13,14,15], and the references therein.

Recently, fractional calculus has become an emerging tool in numerous fields of science and technology. The concept of fractional derivatives generalizes the classical definitions of integer-order derivatives and integrals [16,17]. Due to the hereditary and memory characteristics of fractional derivatives, many real world processes and phenomena are better described by fractional-order models, such as system identification of thermal dynamics of buildings, entropy and information [18,19,20,21]. In addition, the dynamics, chaotic behavior, stability and synchronization of numerous fractional-order neural network models have been investigated in the recent literature [22,23,24,25], including the behavior of fractional impulsive neural networks [26,27,28,29,30]. Besides the most applicable Riemann-Liouville and Caputo types of fractional derivatives, many new types of fractional derivatives were introduced by the researchers. See, for example [31,32,33,34,35], and the references therein.

Despite the great opportunities for applications in modeling of real-world processes, the use of all these derivatives leads to computational complexities. For example, the Riemann–Liouville and Caputo derivatives do not obey the Leibniz rule and chain rule. To overcome these difficulties, new concepts has been proposed in [36,37,38,39,40]. Furthermore, important notes about some of the new concepts are given, for example, in [41,42,43,44,45] and the references therein.

In our early paper [46], we introduced the notion of "fractional-like derivative“ (FLD) which offers some computational simplifications related to FLDs of compositions of functions. Since then, the interest of the researchers to the theory of equations with FLDs has begun. Some basic results on the fundamental and qualitative theory of such equations has been established very recently. See, for example [47,48,49,50] and some of the references therein.

However, the theory of impulsive equations with FLDs is still in a very initial stage. The first results on impulsive equations with conformable derivatives and FLDs have been derived in [51,52,53], where some generalizations of FLDs and integrals have been introduced. Due to the computational convenience that offer generalized FLDs, the theory of such equations needs more developments. Furthermore, the theory of impulsive systems with generalized FLDs has not been applied to real-world models of diverse interest.

The first aim of the present research is to introduce a design of impulsive fractional-like neural network models. The second contribution of our paper is to present efficient stability conditions to the model under consideration. To this end, we investigate its practical stability behavior with respect to manifolds.

It is well known that the stability properties of a neural network are essential for its performance. Furthermore, in numerous cases the model can be unstable in the classical Lyapunov’s sense, but its performance may be sufficient for the practical point of view. For such situations, when the dynamic of systems contained within particular bounds during a fixed time interval, the researchers introduced the notion of practical stability [54,55,56,57]. Due to the great opportunities for applications, the notion has been considered for fractional-order systems [58,59]. For impulsive systems with FLDs, the concept has been investigated only in the paper [52]. However, to the best of the authors’ knowledge, practical stability results have not been derived for impulsive fractional-like neural network systems.

In addition, we will consider the practical stability properties of the designed neural network model with FLDs with respect to manifolds [60,61,62]. Thus, our results are more general than stability (practical stability) results for single solutions: zero solutions, equilibrium, periodic solutions, etc. The case when the behavior of the neural network is affected by some uncertain parameters will also be discussed. Indeed, considering parameters with uncertain values is very important for its qualitative properties [63,64,65].

The rest of the paper is organized as follows. In Section 2, some main definitions and lemmas on generalized FLDs and integrals are presented. We propose a design of an impulsive neural network model with generalized FLDs in Section 3. Some preliminaries are also given. In Section 4, we apply the elaborated in [52] definition of FLDs of piecewise continuous Lyapunov-type functions to derive sufficient conditions for practical stability with respect to manifolds defined by functions. The obtained results are also applied to an impulsive Hopfield fractional-like BAM neural network. In addition, two examples are also presented. Section 5 is devoted to practical stability results for impulsive neural networks with FLDs and uncertain parameters. Finally, the paper concludes in Section 6.

## 2. Generalized FLDs and Integrals

In this Section, we will state some main definitions and lemmas following [51,52,53].

Let $R_{+}=\left[0,\infty \right)$, $R^{n}$ be the *n*-dimensional Euclidean space, and let $\Omega \subset R^{n}$ be a bounded domain that contains the origin.

For given $\tilde{t}\in R_{+}$ and 0<q≤1, we will consider a generalized $q^{th}-$order fractional-like derivative $D_{\tilde{t}}^{q}x\left(t\right)$ for a function $x:\left[\tilde{t},\infty \right)\to R^{n}$ defined as [52]

$$D_{\tilde{t}}^{q}x\left(t\right)=\lim\left\{\frac{x\left(t+\theta {\left(t-\tilde{t}\right)}^{1-q}\right)-x\left(t\right)}{\theta },\;\theta \to 0\right\},\;t>\tilde{t}.$$

Let $t_{0}\in R_{+}$, $t_{0}<t_{1}<t_{2}\ldots <t_{k}<t_{k+1}<\ldots$ and $\lim_{k\to \infty }t_{k}=\infty$. According to [51,52,53]:

- if $\tilde{t}=t_{0}$, then $D_{\tilde{t}}^{q}\left(x\left(t\right)\right)$ has the form
  $$D_{t_{0}}^{q}x\left(t\right)=\lim\left\{\frac{x\left(t+\theta {\left(t-t_{0}\right)}^{1-q}\right)-x\left(t\right)}{\theta },\;\theta \to 0\right\}$$
  which has been applied in systems without impulsive perturbations [36,37,38,39,46,47,48,49,50];
- if $\tilde{t}=0$, then $D_{\tilde{t}}^{q}\left(x\left(t\right)\right)$ has the form
  $$D_{0}^{q}x\left(t\right)=\lim\left\{\frac{x\left(t+\theta t^{1-q}\right)-x\left(t\right)}{\theta },\;\theta \to 0\right\};$$
- if $\tilde{t}=t_{k}$ for some k=1,2,…, then $D_{\tilde{t}}^{q}\left(x\left(\tilde{t}\right)\right)$ has the form
  $$D_{t_{k}}^{q}x\left(t_{k}\right)=\lim_{t\to t_{k}^{+}}D_{t_{k}}^{q}x\left(t\right).$$

If the generalized fractional-like derivative $D_{\tilde{t}}^{q}x\left(t\right)$ of order *q* of a continuous function x(t) exists at any point of an open interval of the type $\left(\tilde{t},b\right)$ for some $b>\tilde{t}$, $t>\tilde{t}$, $\tilde{t}\in R_{+}$, then we will say that the function x(t) is *q*-differentiable on $\left(\tilde{t},b\right)$. The class of all *q*-differentiable on $\left(\tilde{t},b\right)$ functions will be denoted by $C^{q}\left(\left(\tilde{t},b\right),R^{n}\right)$.

Analogous to above, the generalized fractional-like integral of order 0<q≤1 with a lower limit $\tilde{t},\;\tilde{t}\geq 0$, of a function $x:\left[\tilde{t},\infty \right)\to R^{n}$ is defined by (see [52])

$$I_{\tilde{t}}^{q}x\left(t\right)=\int_{\tilde{t}}^{t}{\left(s-\tilde{t}\right)}^{q-1}x\left(s\right)ds.$$

Throughout this paper, we will use the following properties of the generalized FLDs $D_{\tilde{t}}^{q}x\left(t\right)$, $t>\tilde{t}$ for some $\tilde{t}\in R_{+}$ [52].

**Lemma** **1.** *Let $l\left(y\left(t\right)\right):\left(\tilde{t},\infty \right)\to R$. If l(·) is differentiable with respect to y(t) and y(t) is q-differentiable on $\left(\tilde{t},\infty \right)$, where 0<q≤1, then for any $t\in R_{+}$, $t\neq \tilde{t}$ and y(t)≠0*

$$D_{\tilde{t}}^{q}l\left(y\left(t\right)\right)=l^{\prime }\left(y\left(t\right)\right)D_{\tilde{t}}^{q}y\left(t\right),$$

*where $l^{\prime }$ is a partial derivative of l(·).*

**Lemma** **2.** *Let the function $x\left(t\right):\left(\tilde{t},\infty \right)\to R$ be q-differentiable for 0<q≤1. Then for all $t>\tilde{t}$*

$$I_{\tilde{t}}^{q}\left(D_{\tilde{t}}^{q}x\left(t\right)\right)=x\left(t\right)-x\left(\tilde{t}\right).$$

**Remark** **1.** *For more results on FLDs and integrals we refer the reader to [36,37,38,39,46,47,48,49,50], and for results on the generalized FLDs and integrals see [51,52,53].*

## 3. Impulsive Fractional-Like Neural Networks: Main Notions and Definitions

In this paper, we consider the next system of impulsive Hopfield fractional-like neural networks defined as

(1) $$\left\{\begin{array}{c} D_{t_{k}}^{q}x_{i}\left(t\right)=-\frac{1}{C_{i}\left(t\right)R_{i}\left(t\right)}x_{i}\left(t\right)+\sum_{j=1}^{n}\alpha_{ij}\left(t\right)f_{j}\left(x_{j}\left(t\right)\right)+\gamma_{i}\left(t\right),\;t\neq t_{k},\;k=0,1,\ldots ,\\ \Delta x_{i}\left(t_{k}\right)=x_{i}\left(t_{k}^{+}\right)-x_{i}\left(t_{k}\right)=I_{ik}\left(x_{i}\left(t_{k}\right)\right),\;k=1,2,\ldots , \end{array}\right.$$

where $x\left(t\right)=col(x_{1}\left(t\right),x_{2}\left(t\right),\ldots ,x_{n}\left(t\right))$, x∈Ω, $C_{i},R_{i}\in C^{q}\left(R_{+},\left(0,\infty \right)\right)$, $\alpha_{ij}$, $\gamma_{i}\in C^{q}\left(R_{+},R\right)$, $f_{j}\in C^{q}\left(R,R\right)$, $x_{i}\left(t_{k}^{+}\right)=\lim_{h\to 0^{+}}x_{i}\left(t_{k}+h\right)$, $x_{i}\left(t_{k}^{-}\right)=x_{i}\left(t_{k}\right)$, $I_{ik}\in C\left(R,R\right)$, i,j=1,2…,n, n≥2, k=1,2,….

In the above impulsive fractional-like neural network model, $x_{i}\left(t\right)$ represents the state of the i−th node at time *t*, *n* corresponds to the number of neurons in the neural network, the positive functions $C_{i},R_{i}$ are, respectively, the capacitance and the resistance for the node *i* at time *t*, $\alpha_{ij}$ are the connection weights, $f_{j}$ denotes the activation function which determines the output $f_{j}\left(x_{j}\left(t\right)\right)$ of the *j*th unit at time *t*, $\gamma_{i}$ denotes the external bias of the node *i* at time *t*, and $t_{k}$, k=1,2,… are the moments of impulsive perturbations and satisfy $t_{0}<t_{1}<t_{2}\ldots <t_{k}<t_{k+1}<\ldots$, $\lim_{k\to \infty }t_{k}=\infty$. The numbers $x_{i}\left(t_{k}^{-}\right)$ and $x_{i}\left(t_{k}^{+}\right)$ are, respectively, the states of the *i*th node before and after an impulse perturbation at the moment $t_{k}$ and the functions $I_{ik}$ represent the magnitudes of the impulsive changes of the states $x_{i}\left(t\right)$ at the impulsive moments $t_{k}$.

**Remark** **2.** *The designed impulsive fractional-like neural network model generalizes many existing integer-order neural networks [1,2,3,4,8,9,10,11,12,13,14,15]. The main advantages of the proposed model are in (i) incorporating of the hereditary and memory characteristics of fractional derivatives [26,27,28,29,30]; (ii) using the computational simplicity of the generalized FLDs and integrals; (iii) taking into account the effects of some impulsive perturbations that can be used as controls of the neural network’s performance.*

Let $x_{0}=col\left(x_{01},x_{02},\ldots ,x_{0n}\right)\in \Omega$. We will denote by $x\left(t\right)=x\left(t;t_{0},x_{0}\right)$ the solution of the fractional-like impulsive neural network system (1) that satisfies the initial condition

(2) $$x_{i}\left(t_{0}^{+}\right)=x_{0i},\;i=1,2,\ldots ,n.$$

Following the theory of impulsive fractional-order neural network systems [27,30], and the new theory of impulsive fractional-like systems [51,52,53], the solutions x(t) of the neural network models (1) are piecewise continuous functions that have points of discontinuity of the first kind $t_{k}$ and are left continuous at these moments. For such functions, the following identities are satisfied:

$$x_{i}\left(t_{k}^{-}\right)=x_{i}\left(t_{k}\right),\;x_{i}\left(t_{k}^{+}\right)=x_{i}\left(t_{k}\right)+I_{ik}\left(x_{i}\left(t_{k}\right)\right),\;i=1,2,\ldots ,n,\;k=1,2,\ldots .$$

All of these piecewise continuous functions formed the space $PC^{q}\left(R_{+},R^{n}\right)$.

Let $h:[t_{0},\infty )\times \Omega \to R$ be a continuous function. The next sets will be called h−manifolds defined by the function *h*:

$$M_{t}=\left\{x\in \Omega :\;h\left(t,x\right)=0,\;t\in \left[t_{0},\infty \right)\right\},$$

$$M_{t}\left(\varepsilon \right)=\{x\in \Omega :\;|h\left(t,x\right)|<\varepsilon ,\;t\in \left[t_{0},\infty \right)\},\;\varepsilon >0.$$

To guarantee that the solution $x(t;t_{0},x_{0})$ of the initial value problem (IVP) (1)–(2) exists on $\left[t_{0},\infty \right)$, and for the future investigations we will need the following assumptions.

A1. The function *h* is continuous on $[t_{0},\infty )\times \Omega$ and the sets $M_{t}$, $M_{t}\left(\varepsilon \right)$ are (n−1)-dimensional manifolds in $R^{n}$.

A2. Each solution $x(t;t_{0},x_{0})$ of the IVP (1)-(2) satisfying

$$|h(t,x\left(t;t_{0},x_{0}\right))|\leq H<\infty$$

is defined on the interval $\left[t_{0},\infty \right)$, H=const>0.

A3. There exist constants $L_{j}>0$ such that

$$|f_{j}\left(u\right)-f_{j}\left(v\right)|\leq L_{j}\left|u-v\right|,\;f_{j}\left(0\right)=0$$

for all u,v∈R, j=1,2,…,n.

In this paper we will use the following definition for practical exponential stability of the neural network system (1) with respect to manifolds defined by the function *h* given in [51].

**Definition** **1.** *The fractional-like impulsive system (1) is:*

*(a) (λ,A)-practically exponentially stable with respect to the function h, if given (λ,A) with 0<λ<A for any $x_{0}\in M_{t_{0}^{+}}\left(\lambda \right)$ we have*

$$x\left(t;t_{0},x_{0}\right)\in M_{t}(A+\mu |h\left(t_{0}^{+},x_{0}\right)\left|E_{q}\left(-\kappa ,t-t_{0}\right)\right),\;t\geq t_{0}\;for\;some\;t_{0}\in R_{+},$$

*where 0<q<1, μ,κ>0;*

*(b) (λ,A)-globally practically exponentially stable with respect to the function h, if (a) holds for $\Omega \equiv R^{n}.$*

**Remark** **3.** *The problems of exponential stability of integer-order neural networks have been investigated by numerous authors [3,4,8,11,12,13,14]. Indeed, the concept of exponential stability is one of the the most important qualitative concepts for such models because it guarantees the fast convergent rate [13]. The notion of exponential stability has been generalized in [66] to this of Mittag–Leffler stability for fractional-order systems. For Mittag–Leffler stability results of fractional neural networks see, for example [27,28,30] and the bibliography therein. With the present research, we will complement the existing results and will present results on (λ,A)-practical exponential stability for impulsive fractional-like neural network systems.*

## 4. Practical Stability of Impulsive Fractional-Like Neural Networks

### 4.1. Main Practical Stability Results

In this Section, we will state our main practical exponential stability results. Since we consider impulsive effects in the designed neural network model, we will use the following sets

$$G_{k}=\left(t_{k-1},t_{k}\right)\times \Omega ,\;k=1,2,\ldots ,\;G=\overset{\infty }{\underset{k=1}{⋃}}G_{k},$$

and piecewise continuous auxiliary functions [8,9,10,11,12,13,14,15,26,27,28,29,30,52].

What follows is the definition of the class $V_{t_{k}}^{q}$ of Lyapunov-like functions defined in [52] for any $t_{k}\in R_{+}$, k=0,1,2,….

**Definition** **2.** *The function $V\in V_{t_{k}}^{q}$, if:*

1. *V is defined on G, V has non-negative values and V(t,0)=0 for $t\geq t_{k}$;*
2. *V is continuous in G, q−differentiable in t and locally Lipschitz continuous with respect to its second argument on each of the sets $G_{k}$;*
3. *For each k=0,1,2,… and x∈Ω, there exist the finite limits*
  $$V\left(t_{k}^{-},x\right)=\lim_{\overset{t\to t_{k}}{t<t_{k}}}V\left(t,x\right),\;V\left(t_{k}^{+},x\right)=\lim_{\overset{t\to t_{k}}{t>t_{k}}}V\left(t,x\right),$$
  *and $V\left(t_{k}^{-},x\right)=V\left(t_{k},x\right)$.*

For a function $V\in V_{t_{k}}^{q}$, $t>t_{k}$, we define its the upper right fractional-like derivative as [52]:

(3) $$\begin{array}{c} {}^{+}D_{t_{k}}^{q}\;V\left(t,x\right)\\ =lim sup\left\{\frac{V\left(t+\theta {\left(t-t_{k}\right)}^{1-q},\;x\left(t+\theta {\left(t-t_{k}\right)}^{1-q};t,x\right)\right)-V\left(t,x\right)}{\theta },\;\theta \to 0^{+}\right\}. \end{array}$$

Let for simplicity denote by $F\left(t,x\right)=(F_{1}\left(t,x\right),F_{2}\left(t,x\right),\ldots ,F_{n}\left(t,x\right))$, where

$$F_{i}\left(t,x\right)=-\frac{1}{C_{i}\left(t\right)R_{i}\left(t\right)}x_{i}\left(t\right)+\sum_{j=1}^{n}\alpha_{ij}\left(t\right)f_{j}\left(x_{j}\left(t\right)\right)+\gamma_{i}\left(t\right),\;i=1,2,\ldots ,n.$$

Then [46,52] the fractional-like derivative of the function V(t,x) with respect to the solution x(t) of the IVP (1)–(2) is defined by

(4) $$\begin{array}{c} {}^{+}D_{t_{k}}^{q}\;V\left(t,x\right)\\ =\mathrm{lim sup}\left\{\frac{V\left(t+\theta {\left(t-t_{k}\right)}^{1-q},\;x+\theta {\left(t-t_{k}\right)}^{1-q}F\left(t,x\right)\right)-V\left(t,x\right)}{\theta },\;\theta \to 0^{+}\right\}. \end{array}$$

If V(t,x(t))=V(x(t)),0<q≤1, *V* is differentiable on *x*, and x(t) is *q*-differentiable on *t* for $t>t_{k}$, then

$${}^{+}D_{t_{k}}^{q}\;V\left(t,x\right)=V^{\prime }\left(x\left(t\right)\right)\;D_{t_{k}}^{q}x\left(t\right),$$

where $V^{\prime }$ is a partial derivative of the function *V*.

From (3) and (4) it follows

$${}^{+}D_{t_{k}}^{q}\;V\left(t,x\left(t;t_{0},x_{0}\right)\right)=\;{\;}^{+}D_{t_{k}}^{q}\;V\left(t,x\right)\;{|}_{\left(1\right)},$$

$t>t_{k}$, k=0,1,2,….

We will also need the following result from [52].

**Lemma** **3.** *Assume that the function $V\in V_{t_{k}}^{q}$ is such that for $t\in [t_{0},\infty )$, x∈Ω,*

$$V\left(t_{k}^{+},x\right)\leq V\left(t_{k},x\right),\;k=1,2,\ldots ,$$

$${}^{+}D_{t_{k}}^{q}\;V\left(t,x\right)\leq -\kappa V\left(t,x\right)+g\left(t\right),\;t\neq t_{k},\;k=0,1,2,\ldots ,$$

*where $\kappa =const>0,\;g\in \;C^{q}\left(R_{+},R_{+}\right)$.*

*Then*

$$V\left(t,x\left(t\right)\right)\leq V\left(t_{0}^{+},x_{0}\right)E_{q}\left(-\kappa ,t-t_{0}\right)+\int_{t_{k}}^{t}\frac{W^{q}\left(t-t_{k},s-t_{k}\right)g\left(s\right)}{{\left(s-t_{k}\right)}^{1-q}}ds$$

$$+\sum_{j=1}^{k}\;\prod_{l=k-j+1}^{k}E_{q}\left(-\kappa ,t_{l}-t_{l-1}\right)\int_{t_{k-j}}^{t_{k-j+1}}\frac{W^{q}\left(t-t_{k},s-t_{k-j}\right)g\left(s\right)}{{\left(s-t_{k_{j}}\right)}^{1-q}}ds,\;t\geq t_{0},$$

*where $W^{q}\left(t-t_{k},s-t_{k}\right)=E_{q}\left(-\kappa ,t-t_{k}\right)E_{q}\left(\kappa ,s-t_{k}\right)$ and $E_{q}\left(\nu ,s\right)$ is the fractional-like exponential function defined as [37,39]*

$$E_{q}\left(\nu ,s\right)=\exp\left(\nu \frac{s^{q}}{q}\right),\;\nu \in R,s\in R_{+}.$$

In what follows, for a bounded continuous function *f* defined on $R_{+}$, we set

$$\bar{f}=\underset{t\in R_{+}}{\sup}f\left(t\right),\;\underline{f}=\underset{t\in R_{+}}{\inf}f\left(t\right).$$

**Theorem** **1.** *Assume that 0<λ<A are given, and:*

*1. Assumptions A1–A3 hold.*

*2. The models’ parameters $C_{i}$, $R_{i}$ and $\alpha_{ij}$, i,j=1,2,…,n, satisfy*

$$\min_{1\leq i\leq n}\frac{1}{{\bar{C}}_{i}{\bar{R}}_{i}}>\max_{1\leq i\leq n}\left(L_{i}\sum_{j=1}^{n}\left|{\bar{\alpha }}_{ji}\right|\right)$$

and $\kappa^{*}>0$ is such that

$$\min_{1\leq i\leq n}\frac{1}{{\bar{C}}_{i}{\bar{R}}_{i}}-\max_{1\leq i\leq n}\left(L_{i}\sum_{j=1}^{n}\left|{\bar{\alpha }}_{ji}\right|\right)\geq \kappa^{*}>0.$$

*3. For $t\geq t_{0}$ the system’s parameters $\gamma_{i}$, i=1,2,…,n, satisfy*

$$g\left(t\right)=\int_{t_{0}}^{\infty }\frac{W^{q}\left(t-t_{k},s-t_{k}\right)}{{\left(s-t_{0}\right)}^{1-q}}\sum_{i=1}^{n}\left|\gamma_{i}\left(s\right)\right|ds$$

$$+\sum_{j=1}^{k}\;\prod_{l=k-j+1}^{k}E_{q}\left(-\kappa^{*},t_{l}-t_{l-1}\right)\int_{t_{k-j}}^{t_{k-j+1}}\frac{W^{q}\left(t-t_{k},s-t_{k-j}\right)}{{\left(s-t_{k_{j}}\right)}^{1-q}}\sum_{i=1}^{n}\left|\gamma_{i}\left(s\right)\right|ds<\infty .$$

*4. The functions $I_{k}=diag\left(I_{1k},I_{2k},\ldots ,I_{nk}\right)$ are such that*

$$I_{ik}\left(x_{i}\left(t_{k}\right)\right)=-\gamma_{ik}x_{i}\left(t_{k}\right),\;0<\gamma_{ik}<2,\;i=1,2,\ldots n,\;k=1,2,\ldots$$

*and x∈Ω implies $x+I_{k}\left(x\right)\in \Omega$ for k=1,2,….*

*5. The function h(t,x) satisfies*

$$|h\left(t,x\right)|<\sum_{i=1}^{n}|x_{i}\left(t\right)|\leq \Lambda \left(H\right)|h\left(t,x\right)|,\;t\in \left[t_{0},\infty \right),$$

*where Λ(H)≥1 exists for any 0<H≤∞.*

*Then the neural network system (1) is (λ,A)-practically exponentially stable with respect to the function h.*

**Proof.**  Let

$$x\left(t\right)=(x_{1}{\left(t,x_{2}\left(t\right),\ldots ,x_{n}\left(t\right)\right)}^{T}$$

be a solution of (1) for $x_{0}\in \Omega$.

Consider the Lyapunov-like function

$$V\left(x\left(t\right)\right)=\sum_{i=1}^{n}\left|x_{i}\left(t\right)\right|.$$

We can easily check that $V\in V_{t_{k}}^{q}$. For $t_{k}>t_{0}\geq 0$, k=1,2,…, from condition 4 of Theorem 1 we have that $x(t_{k})\in \Omega$ implies $x(t_{k}^{+})\in \Omega$ for k=1,2,…, and

(5) $$V\left(x\left(t_{k}^{+}\right)\right)=\sum_{i=1}^{n}|x_{i}\left(t_{k}^{+}\right)|=\sum_{i=1}^{n}\left|\left(1-\gamma_{ik}\right)x_{i}\left(t_{k}\right)\right|\leq V\left(x\left(t_{k}\right)\right).$$

From A3 for $t\in (t_{k},t_{k+1}]$, k=0,1,2,…, we get

$${}^{+}D_{t_{k}}^{q}\;V\left(x\left(t\right)\right)\leq -\sum_{i=1}^{n}\frac{1}{C_{i}\left(t\right)R_{i}\left(t\right)}|x_{i}\left(t\right)|+\sum_{i=1}^{n}\sum_{j=1}^{n}|\alpha_{ij}\left(t\right)||f_{j}\left(x_{j}\left(t\right)\right)|+\sum_{i=1}^{n}\left|\gamma_{i}\left(t\right)\right|$$

$$\leq -\sum_{i=1}^{n}\frac{1}{{\bar{C}}_{i}{\bar{R}}_{i}}|x_{i}\left(t\right)|+\sum_{i=1}^{n}\sum_{j=1}^{n}|{\bar{\alpha }}_{ij}\left(t\right)|L_{j}|x_{j}\left(t\right)|+\sum_{i=1}^{n}\left|\gamma_{i}\left(t\right)\right|$$

$$\leq -\min_{1\leq i\leq n}\frac{1}{{\bar{C}}_{i}{\bar{R}}_{i}}\sum_{i=1}^{n}|x_{i}\left(t\right)|+\max_{1\leq i\leq n}\left(L_{i}\sum_{j=1}^{n}\left|{\bar{\alpha }}_{ji}\right|\right)\sum_{i=1}^{n}|x_{i}\left(t\right)|+\sum_{i=1}^{n}\left|\gamma_{i}\left(t\right)\right|$$

$$=-\left(\kappa_{1}-\kappa_{2}\right)V\left(x\left(t\right)\right)+\sum_{i=1}^{n}\left|\gamma_{i}\left(t\right)\right|,$$

where

$$\kappa_{1}=\min_{1\leq i\leq n}\frac{1}{{\bar{C}}_{i}{\bar{R}}_{i}},\;\kappa_{2}=\max_{1\leq i\leq n}\left(L_{i}\sum_{j=1}^{n}\left|{\bar{\alpha }}_{ji}\right|\right).$$

From condition 2 of Theorem 1, it follows that there exits a real number $\kappa^{*}>0$ such that

$$\kappa_{1}-\kappa_{2}\geq \kappa^{*}$$

and for $t\in (t_{k},t_{k+1}]$, k=0,1,2,…, along (1) we obtain

(6) $${}^{+}D_{t_{k}}^{q}\;V\left(x\left(t\right)\right)\leq -\kappa^{*}V\left(x\left(t\right)\right)+\sum_{i=1}^{n}\left|\gamma_{i}\left(t\right)\right|.$$

From the last inequality, (5) and Lemma 1 we get

(7) $$\begin{array}{c} V\left(x\left(t\right)\right)\leq V\left(x\left(t_{0}^{+}\right)\right)E_{q}\left(-\kappa^{*},t-t_{0}\right)+\int_{t_{0}}^{\infty }\frac{W^{q}\left(t-t_{k},s-t_{k}\right)}{{\left(s-t_{0}\right)}^{1-q}}\sum_{i=1}^{n}\left|\gamma_{i}\left(s\right)\right|ds\\ +\sum_{j=1}^{k}\;\prod_{l=k-j+1}^{k}E_{q}\left(-\kappa^{*},t_{l}-t_{l-1}\right)\int_{t_{k-j}}^{t_{k-j+1}}\frac{W^{q}\left(t-t_{k},s-t_{k-j}\right)}{{\left(s-t_{k_{j}}\right)}^{1-q}}\sum_{i=1}^{n}\left|\gamma_{i}\left(s\right)\right|ds. \end{array}$$

Let $x_{0}\in M_{t_{0}^{+}}\left(\lambda \right)$, i.e., $|h(t_{0}^{+},x_{0})|<\lambda$. Then from condition 3 of Theorem 3 it follows that can choose *A* so that g(t)<A.

From (7) and condition 5 of Theorem 1 we obtain

$$|h\left(t,x\left(t;t_{0},x_{0}\right)\right)|<V\left(x\left(t;t_{0},x_{0}\right)\right)\leq A+\Lambda \left(H\right)\left|h\left(t_{0}^{+},x_{0}\right)\right|E_{q}\left(-\kappa^{*},t-t_{0}\right),\;t\geq t_{0}.$$

Therefore,

$$x\left(t;t_{0},x_{0}\right)\in M_{t}\left(A+\Lambda \left(H\right)|h\left(t_{0}^{+},x_{0}\right)|E_{q}\left(-\kappa^{*},t-t_{0}\right)\right)$$

for $t\geq t_{0}$, i.e., the system (1) is (λ,A)-practically exponentially stable with respect to the function *h*. □

**Remark** **4.** *If the assumptions of Theorem 1 hold globally on $R^{n}$, i.e., if $\Omega \equiv R^{n}$, then the system (1) is (λ,A)-globally practically exponentially stable with respect to the function h. Note that, in this case the condition x∈Ω implies $x+I_{k}\left(x\right)\in \Omega$ for k=1,2,… is obvious.*

**Remark** **5.** Theorem 1 offers sufficient conditions for practical exponential stability (global practical exponential stability) with respect to a function h for the designed fractional-like impulsive neural network model. Exponential stability results for single solutions of the model (1) (equilibrium, zero solution, periodic solution) can be obtained as corollaries for particular choices of the function h. For example, in the case when $h\left(t,x\right)=||x-x^{*}\left|\right|$, where $x^{*}$ is a single solution of (1) and ||.|| is the norm in $R^{n}$, our results extend and improve the existing exponential stability results for integer-order neural networks [3,4,8,11,12,13,14].

**Remark** **6.** *Our results also complement the existing Mittag–Leffler stability results for fractional neural networks [27,28,30]. The key features of FLDs provide less complicated from the computational aspects criteria. Thus, the new results are more appropriate for the numerous applications of neural network models with derivatives of non integer order.*

The new exponential stability results proved in Theorem 1 can be useful for various classes of fractional-like neural network models. Next, we will apply the obtained criteria to study the practical stability properties of following system of impulsive Hopfield fractional-like bidirectional associative memory (BAM) neural networks:

(8) $$\left\{\begin{array}{c} D_{t_{k}}^{q}y_{i}\left(t\right)=-\frac{1}{C_{i}^{y}R_{i}^{y}}y_{i}\left(t\right)+\sum_{j=1}^{n_{1}}w_{ji}f_{j}^{z}\left(z_{j}\left(t\right)\right)+\gamma_{i}^{y}\left(t\right),\\ D_{t_{k}}^{q}z_{j}\left(t\right)=-\frac{1}{C_{j}^{z}R_{j}^{z}}z_{j}\left(t\right)+\sum_{i=1}^{n_{2}}h_{ij}g_{i}^{y}\left(y_{i}\left(t\right)\right)+\gamma_{j}^{z}\left(t\right),\;t\neq t_{k},\;k=0,1,2,\ldots ,\\ \Delta y_{i}\left(t_{k}\right)=Q_{ik}y_{i}\left(t_{k}\right),\\ \Delta z_{j}\left(t_{k}\right)=T_{jk}z_{j}\left(t_{k}\right),\;k=1,2,\ldots , \end{array}\right.$$

where $t_{0}\in R_{+},\;t_{0}<t_{1}<t_{2},\ldots$, $j=1,2,\ldots ,n_{1}$, $i=1,2,\ldots ,n_{2}$, $n=n_{1}+n_{2}$, $x_{i}\left(t\right)$ and $y_{j}\left(t\right)$ correspond to the states of the *i*th unit and *j*th unit, respectively, at time *t*, $C_{i}^{y},R_{i}^{y}$, $C_{i}^{z},R_{i}^{z}$ are positive constants, the real constants $w_{ji}$, $h_{ij}$ are the connection weights, $f_{j}^{z},g_{i}^{y}\in C^{q}\left[R,R\right]$ are the activation functions; $\gamma_{i}^{y},\gamma_{j}^{z}\in C^{q}\left[R_{+},R\right]$ denote external inputs at time *t*, and the constants $Q_{ik}$, $T_{jk}$ determine the abrupt changes of the states at the impulsive moments $t_{k}$.

Note that different types of BAM neural networks of integer order have been intensively investigated due to the great opportunities for their application in many fields such as pattern recognition and automatic control [11,12]. Results on fractional BAM neural network models with Caputo fractional derivatives have been also published in the recent literature. See, for example [27] and the references therein. In this Section, we will extend the existing results to the fractional-like case.

Let $t_{0}\in R_{+}$ and $y_{0}\in R^{n_{2}}$, $z_{0}\in R^{n_{1}}$. Denote by

$${\left(y\left(t\right),z\left(t\right)\right)}^{T}={\left(y_{1}\left(t\right),\ldots ,y_{n_{2}}\left(t\right),z_{1}\left(t\right),\ldots ,z_{n_{1}}\left(t\right)\right)}^{T}\in R^{n}$$

the solution of system (8) satisfying the initial conditions:

$$\left\{\begin{array}{c} y\left(t_{0}^{+};t_{0},y_{0}\right)=y_{0},\\ z\left(t_{0}^{+};t_{0},z_{0}\right)=z_{0}. \end{array}\right.$$

We introduce the following conditions:

A4. There exist constants $L_{j}^{z}>0$ and $M_{i}^{y}>0$ such that

$$|f_{j}^{z}\left(u\right)-f_{j}^{z}\left(v\right)|\leq L_{j}^{z}|u-v|,\;f_{j}^{z}\left(0\right)=0,\;|g_{i}^{y}\left(u\right)-g_{i}^{y}\left(v\right)|\leq M_{i}^{y}\left|u-v\right|,\;g_{i}^{y}\left(0\right)=0$$

for all u,v∈R, $j=1,2,\ldots ,n_{1},\;i=1,2,\ldots ,n_{2}$.

A5. The constants $Q_{ik}$ and $T_{jk}$ are such that

$$-2<Q_{ik}<0,\;\;-2<T_{jk}<0$$

for $j=1,2,\ldots ,n_{1},\;i=1,2,\ldots ,n_{2},\;k=1,2,\ldots$.

The next result follows directly from Theorem 1.

**Theorem** **2.** *Assume that 0<λ<A are given, and:*

*1. Assumptions A1, A2, A4, A5 hold.*

*2. For $j=1,2,\ldots ,n_{1},\;i=1,2,\ldots ,n_{2}$ it follows*

$$\max_{1\leq i\leq n_{2}}\left(M_{i}^{y}\sum_{j=1}^{n_{1}}\left|h_{ij}\right|\right)<\min_{1\leq i\leq n_{2}}\frac{1}{C_{i}^{y}R_{i}^{y}},\;\max_{1\leq j\leq n_{1}}\left(L_{j}^{z}\sum_{i=1}^{n_{2}}\left|w_{ji}\right|\right)<\min_{1\leq j\leq n_{1}}\frac{1}{C_{j}^{z}R_{j}^{z}}$$

and $\kappa^{*}$ is such that

$$0<\kappa^{*}\leq \min\left\{\min_{1\leq i\leq n_{2}}\frac{1}{C_{i}^{y}R_{i}^{y}}-\max_{1\leq i\leq n_{2}}\left(M_{i}^{y}\sum_{j=1}^{n_{1}}\left|h_{ij}\right|\right),\min_{1\leq j\leq n_{1}}\frac{1}{C_{j}^{z}R_{j}^{z}}-\max_{1\leq j\leq n_{1}}\left(L_{j}^{z}\sum_{i=1}^{n_{2}}\left|w_{ji}\right|\right)\right\}.$$

*3. For $t\in [t_{0},\infty )$ we have*

$$\bar{G}\left(t\right)=\int_{t_{0}}^{\infty }\frac{W^{q}\left(t-t_{k},s-t_{k}\right)}{{\left(s-t_{0}\right)}^{1-q}}\left(\sum_{i=1}^{n_{2}}|\gamma_{i}^{y}\left(s\right)|+\sum_{j=1}^{n_{1}}\left|\gamma_{j}^{z}\left(s\right)\right|\right)ds$$

$$+\sum_{j=1}^{k}\;\prod_{l=k-j+1}^{k}E_{q}\left(-\kappa^{*},t_{l}-t_{l-1}\right)\int_{t_{k-j}}^{t_{k-j+1}}\frac{W^{q}\left(t-t_{k},s-t_{k-j}\right)}{{\left(s-t_{k_{j}}\right)}^{1-q}}\left(\sum_{i=1}^{n_{2}}|\gamma_{i}^{y}\left(s\right)|+\sum_{j=1}^{n_{1}}\left|\gamma_{j}^{z}\left(s\right)\right|\right)ds<\infty ;$$

*4. For the function h(t,y,z) we have*

$$\left|h\left(t,y,z\right)\right|\leq \sum_{i=1}^{n_{1}}|z_{i}\left(t\right)|+\sum_{j=1}^{n_{2}}|y_{j}\left(t\right)|\leq \Lambda \left(H\right)|h\left(t,y,z\right)|,\;t\in \left[t_{0},\infty \right),$$

*where Λ(H)≥1 exists for any 0<H≤∞.*

*Then (8) is (λ,A)-globally practically exponentially stable with respect to the function h.*

**Proof.**  The proof of Theorem 2 follows the steps in the proof of Theorem 1. In this case we can use the Lyapunov’s function

$$V\left(y\left(t\right),z\left(t\right)\right)=\sum_{i=1}^{n_{1}}|z_{i}\left(t\right)|+\sum_{j=1}^{n_{2}}\left|y_{j}\left(t\right)\right|.$$

Then, inequalities in the form (5) follow from the condition A5 and instead of (7), from condition 1 of Theorem 2, we get

$${}^{+}D_{t_{k}}^{q}\;V\left(y\left(t\right),z\left(t\right)\right)\leq \sum_{j=1}^{n_{1}}\left(\sum_{i=1}^{n_{2}}\left|w_{ji}\right|L_{j}^{z}-\frac{1}{C_{j}^{z}R_{j}^{z}}\right)|z_{j}\left(t\right)|+\sum_{i=1}^{n_{2}}\left(\sum_{j=1}^{n_{1}}\left|h_{ij}\right|M_{i}^{y}-\frac{1}{C_{i}^{y}R_{i}^{y}}\right)\left|y_{i}\left(t\right)\right|$$

$$+\sum_{j=1}^{n_{1}}|\gamma_{j}^{z}\left(t\right)|+\sum_{i=1}^{n_{2}}\left|\gamma_{i}^{y}\left(t\right)\right|.$$

Condition 2 of Theorem 2 implies the existence of a positive number $\kappa^{*}$ such that

$$\kappa^{*}\leq \min\left\{\min_{1\leq i\leq n_{2}}\frac{1}{C_{i}^{y}R_{i}^{y}}-\max_{1\leq i\leq n_{2}}\left(M_{i}^{y}\sum_{j=1}^{n_{1}}\left|h_{ij}\right|\right),\min_{1\leq j\leq n_{1}}\frac{1}{C_{j}^{z}R_{j}^{z}}-\max_{1\leq j\leq n_{1}}\left(L_{j}^{z}\sum_{i=1}^{n_{2}}\left|w_{ji}\right|\right)\right\},$$

and, hence

$${}^{+}D_{t_{k}}^{q}\;V\left(y\left(t\right),z\left(t\right)\right)\leq -\kappa^{*}V\left(y\left(t\right),z\left(t\right)\right)+\bar{G}\left(t\right).$$

The proof is completed by applying conditions 3 and 4 of Theorem 2. □

### 4.2. Examples

**Example** **1.** *Consider the following 2-D impulsive fractional-like Hopfield neural network model*

(9) $$\left\{\begin{array}{c} D_{t_{k}}^{q}x_{i}\left(t\right)=-\frac{1}{C_{i}\left(t\right)R_{i}\left(t\right)}x_{i}\left(t\right)+\sum_{j=1}^{2}\alpha_{ij}\left(t\right)f_{j}\left(x_{j}\left(t\right)\right)+\gamma_{i}\left(t\right),\;t\neq t_{k},\;k=0,1,\ldots ,\\ \Delta x\left(t_{k}\right)=\left(\begin{array}{cc} -\frac{3}{4} & 0\\ 0 & -\frac{2}{3} \end{array}\right)x\left(t_{k}\right),\;k=1,2,\ldots , \end{array}\right.$$

*where i=1,2, $t_{0}=0$,*

$$\begin{array}{c} x\left(t\right)=\left(\begin{array}{c} x_{1}\left(t\right)\\ x_{2}\left(t\right) \end{array}\right),\;\gamma_{1}\left(t\right)=\gamma_{2}\left(t\right)=0,\;C_{1}=e^{-t}/4,\;C_{2}=e^{-t}/3,\\ R_{1}=R_{2}=1,\;f_{j}\left(x_{j}\right)=\frac{|x_{j}+1|-|x_{j}-1|}{2},\;j=1,2,\\ \alpha_{11}\left(t\right)=0.3+\sin\left(t\right),\;\alpha_{12}\left(t\right)=0.1-0.6\cos\left(t\right)-0.4\sin t,\\ \alpha_{21}\left(t\right)=0.3\cos\left(t\right)+0.7,\;\alpha_{22}\left(t\right)=0.8-0.3\cos\left(t\right)+0.2\sin\left(t\right), \end{array}$$

*$0<t_{1}<t_{2}<\ldots$ and $t_{k}\to \infty$ as k→∞.*

*Since*

$$\begin{array}{c} {\bar{\alpha }}_{11}=1.3,\;{\bar{\alpha }}_{12}=1.1,\;{\bar{\alpha }}_{21}=1,\;{\bar{\alpha }}_{22}=1.3,\\ {\bar{C}}_{1}=\frac{1}{4},\;{\bar{C}}_{1}=\frac{1}{3},\;{\bar{R}}_{1}={\bar{R}}_{2}=1, \end{array}$$

then condition 2 of Theorem 1 is satisfied and $0<\kappa^{*}\leq 0.6$.

*Also, for $\sum_{i=1}^{n}\left|\gamma_{i}\left(t\right)\right|=0$, we can choose 0<λ<A so that g(t)<A.*

*In addition, conditions 4 of Theorem 1 is satisfied, since*

$$0<\gamma_{1k}=\frac{3}{4}<2,\;0<\gamma_{2k}=\frac{2}{3}<2,\;k=1,2,\ldots .$$

**Figure 1:**
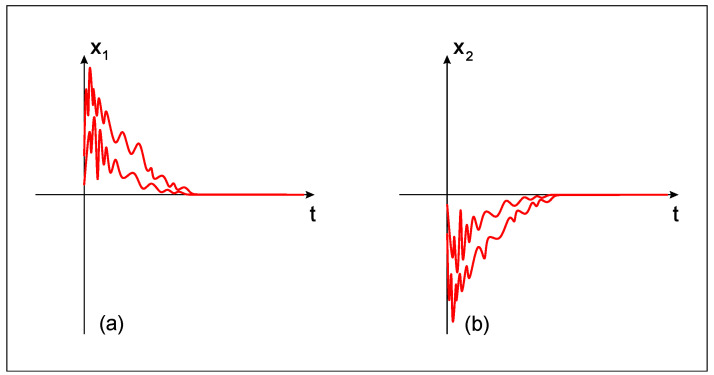
The (λ,A)-global exponentially stable behavior of the fractional-like neural network model (9) with respect to the function $h=|x_{1}\left|+\right|x_{2}|$ for λ=5, A=9. (**a**) Behavior of the state variable $x_{1}\left(t\right)$; (**b**) Behavior of the state variable $x_{2}\left(t\right)$.

*Therefore, according to Theorem 1, the impulsive fractional-like neural network system (9) is (λ,A)-globally practically exponentially stable with respect to the function $h\left(x_{1},x_{2}\right)=|x_{1}\left|+\right|x_{2}|$. The global exponentially stable behavior is shown in Figure 1 for λ=5, A=9.*

**Example** **2.** *Consider the following impulsive BAM fractional-like Hopfield neural network model*

(10) $$\left\{\begin{array}{c} D_{t_{k}}^{q}y_{i}\left(t\right)=-\frac{1}{C_{i}^{y}R_{i}^{y}}y_{i}\left(t\right)+\sum_{j=1}^{2}w_{ji}f_{j}^{z}\left(z_{j}\left(t\right)\right)+\gamma_{i}^{y}\left(t\right),\\ D_{t_{k}}^{q}z_{j}\left(t\right)=-\frac{1}{C_{j}^{z}R_{j}^{z}}z_{j}\left(t\right)+\sum_{i=1}^{2}h_{ij}g_{i}^{y}\left(y_{i}\left(t\right)\right)+\gamma_{j}^{z}\left(t\right),\;t\neq t_{k},\;k=0,1,2,\ldots ,\\ \;\Delta y_{1}\left(t_{k}\right)=Q_{1k}\left(y_{1}\left(t_{k}\right)-2\right),\;\Delta y_{2}\left(t_{k}\right)=Q_{2k}\left(y_{2}\left(t_{k}\right)-1\right),\\ \;\Delta z_{1}\left(t_{k}\right)=T_{1k}\left(z_{1}\left(t_{k}\right)-2\right),\;\Delta z_{2}\left(t_{k}\right)=T_{2k}\left(z_{2}\left(t_{k}\right)-3\right),\;k=1,2,\ldots , \end{array}\right.$$

*where i,j=1,2, $t_{0}=0$, $\gamma_{1}^{y}\left(t\right)=2.2$, $\gamma_{2}^{y}\left(t\right)=3.6$, $\gamma_{1}^{z}\left(t\right)=\gamma_{2}^{z}\left(t\right)=2.8$,*

$$\begin{array}{c} y\left(t\right)=\left(\begin{array}{c} y_{1}\left(t\right)\\ y_{2}\left(t\right) \end{array}\right),\;z\left(t\right)=\left(\begin{array}{c} z_{1}\left(t\right)\\ z_{2}\left(t\right) \end{array}\right),\;C_{1}^{y}=\frac{1}{2},\;C_{2}^{y}=\frac{1}{3},\;C_{1}^{z}=\frac{1}{5},\;C_{2}^{z}=\frac{1}{4},\\ R_{1}^{y}=\frac{4}{3},\;R_{2}^{y}=\frac{3}{4},\;R_{1}^{z}=\frac{5}{2},\;R_{2}^{z}=4,\;f_{j}^{z}\left(z_{j}\right)=\frac{|z_{j}+1|-|z_{j}-1|}{2},\;j=1,2,\\ g_{i}^{y}\left(y_{i}\right)=\frac{|y_{i}+1|-|y_{i}-1|}{2},\;i=1,2,\;w_{11}=0.3\;w_{12}\left(t\right)=0.6,\;w_{21}=0.5,\;w_{22}\left(t\right)=-0.2,\\ h_{11}=0.7,\;w_{12}\left(t\right)=0.5,\;w_{21}=0.3,\;w_{22}\left(t\right)=-0.1,\;Q_{ik}=-\left(1+\frac{1}{2i}\cos\left(2k^{3}\right)\right),\\ T_{jk}=-\left(1+\frac{2}{5j}\sin\left(1+k\right)\right),\;i,j=1,2,\;k=1,2,\ldots ,\;0<t_{1}<t_{2}<\ldots \end{array}$$

*and $t_{k}\to \infty$ as k→∞.*

*We can easily find that the neural network system (10) has an equilibrium*

(11) $${\left(y^{*},z^{*}\right)}^{T}={\left(y_{1}^{*},y_{2}^{*},z_{1}^{*},z_{2}^{*}\right)}^{T}={\left(2,1,2,3\right)}^{T}.$$

Set ${\bar{y}}_{i}=y_{i}-y_{i}^{*}$, ${\bar{z}}_{j}=z_{j}-z_{j}^{*}$, i,j=1,2. Then

(12) $$\left\{\begin{array}{c} D_{t_{k}}^{q}{\bar{y}}_{i}\left(t\right)=-\frac{1}{C_{i}^{y}R_{i}^{y}}{\bar{y}}_{i}\left(t\right)+\sum_{j=1}^{2}w_{ji}\left(f_{j}^{z}\left(z_{j}\left(t\right)\right)-f_{j}^{z}\left(z_{j}^{*}\right)\right),\\ D_{t_{k}}^{q}{\bar{z}}_{j}\left(t\right)=-\frac{1}{C_{j}^{z}R_{j}^{z}}{\bar{z}}_{j}\left(t\right)+\sum_{i=1}^{2}h_{ij}\left(g_{i}^{y}\left(y_{i}\left(t\right)\right)-g_{i}^{y}\left(y_{i}^{*}\right)\right),\;t\neq t_{k},\;k=0,1,2,\ldots ,\\ \;\Delta {\bar{y}}_{1}\left(t_{k}\right)=Q_{1k}{\bar{y}}_{1}\left(t_{k}\right),\;\Delta {\bar{y}}_{2}\left(t_{k}\right)=Q_{2k}{\bar{y}}_{2}\left(t_{k}\right),\\ \;\Delta {\bar{z}}_{1}\left(t_{k}\right)=T_{1k}{\bar{z}}_{1}\left(t_{k}\right),\;\Delta {\bar{z}}_{2}\left(t_{k}\right)=T_{2k}{\bar{z}}_{2}\left(t_{k}\right),\;k=1,2,\ldots . \end{array}\right.$$

*For the system (12) all conditions of Theorem 2 are satisfied. Indeed, we have that $L_{j}^{z}=M_{i}^{y}=1$, i,j=1,2,*

$$1.2=\max_{1\leq i\leq 2}\left(M_{i}^{y}\sum_{j=1}^{2}\left|h_{ij}\right|\right)<\min_{1\leq i\leq 2}\frac{1}{C_{i}^{y}R_{i}^{y}}=1.5,$$

$$0.8=\max_{1\leq j\leq 2}\left(L_{j}^{z}\sum_{i=1}^{2}\left|w_{ji}\right|\right)<\min_{1\leq j\leq 2}\frac{1}{C_{j}^{z}R_{j}^{z}}=1,$$

$$\;-2<Q_{ik}<0,\;\;-2<T_{jk}<0$$

for i,j=1,2,k=1,2,… and $0<\kappa^{*}\leq 0.2$.

**Figure 2:**
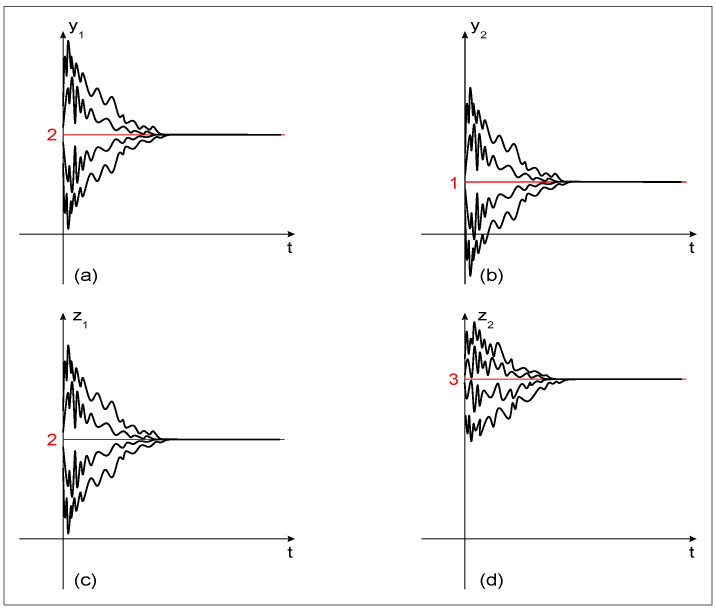
The (λ,A)-global exponentially stable behavior of model (10) with respect to the function $h\left(y_{1},y_{2},z_{1},z_{2}\right)=\sqrt{{\left(y_{1}-y_{1}^{*}\right)}^{2}+{\left(y_{2}-y_{2}^{*}\right)}^{2}+{\left(z_{1}-z_{1}^{*}\right)}^{2}+{\left(z_{2}-z_{2}^{*}\right)}^{2}}$ for λ=8, A=11. (**a**) Behavior of the state variable $y_{1}\left(t\right)$; (**b**) Behavior of the state variable $y_{2}\left(t\right)$; (**c**) Behavior of the state variable $z_{1}\left(t\right)$; (**d**) Behavior of the state variable $z_{2}\left(t\right)$.

Hence, the fractional-like impulsive BAM neural network system (10) is (λ,A)-globally practically exponentially stable with respect to the function $h\left(y_{1},y_{2},z_{1},z_{2}\right)=\sqrt{{\left(y_{1}-y_{1}^{*}\right)}^{2}+{\left(y_{2}-y_{2}^{*}\right)}^{2}+{\left(z_{1}-z_{1}^{*}\right)}^{2}+{\left(z_{2}-z_{2}^{*}\right)}^{2}}$. The global exponentially stable behavior is shown in Figure 2 for λ=8, A=11.

## 5. Impulsive Fractional-Like Neural Networks with Uncertain Parameters

In this Section, we will consider an impulsive neural network system with FLDs and uncertain parameters given by

(13) $$\left\{\begin{array}{c} D_{t_{k}}^{q}x_{i}\left(t\right)=-\left(\frac{1}{C_{i}\left(t\right)R_{i}\left(t\right)}+{\tilde{a}}_{i}\left(t\right)\right)x_{i}\left(t\right)\\ \;\;+\sum_{j=1}^{n}\left(\alpha_{ij}\left(t\right)+{\tilde{\alpha }}_{ij}\left(t\right)\right)f_{j}\left(x_{j}\left(t\right)\right)+\gamma_{i}\left(t\right)+{\tilde{\gamma }}_{i}\left(t\right),\;t\neq t_{k},\;k=0,1,\ldots ,\\ \Delta x_{i}\left(t_{k}\right)=\left(-\gamma_{ik}+{\tilde{P}}_{ik}\right)x_{i}\left(t_{k}\right),\;k=1,2,\ldots , \end{array}\right.$$

where the functions ${\tilde{a}}_{i}\in C^{q}\left[R_{+},\left(0,\infty \right)\right]$, ${\tilde{\alpha }}_{ij},{\tilde{\gamma }}_{i}\in C^{q}\left[R_{+},R\right]$, i,j=1,2,…,n,k=1,2,… and constants ${\tilde{P}}_{ik},\;i=1,2,\ldots ,n,\;k=1,2,\ldots$, represent the uncertainty of the system [63]. In the case when all of these functions and constants are zeros the system (13) will be reduced to the “nominal system” (1). [63,64,65].

**Definition** **3.** *The system (1) is called (λ,A)-practically robustly exponentially stable with respect to the function h if for given (λ,A) with 0<λ<A, $t_{0}\in R_{+}$, $x_{0}\in M_{t_{0}^{+}}\left(\lambda \right)$ and for any ${\tilde{a}}_{i},\;{\tilde{\alpha }}_{ij},\;{\tilde{\gamma }}_{i},\;{\tilde{P}}_{ik},\;i,j=1,2,\ldots ,n,\;k=1,2,\ldots ,$ the system (13) is (λ,A)-practically exponentially stable with respect to the function h.*

Using Theorem 1, we can prove the next result.

**Theorem** **3.** *Assume that:*

*1. Conditions of Theorem 1 hold.*

*2. For i,j=1,2,…,n the functions ${\tilde{\gamma }}_{i}\left(t\right),\;{\tilde{a}}_{i}\left(t\right)$ and ${\tilde{\alpha }}_{ij}\left(t\right)$ are bounded for $t\in [t_{0},\infty )$,*

$$\min_{1\leq i\leq n}\left(\frac{1}{{\bar{C}}_{i}{\bar{R}}_{i}}+{\bar{\tilde{a}}}_{i}\right)>\max_{1\leq i\leq n}\left(L_{i}\sum_{j=1}^{n}\left(\right|{\bar{\alpha }}_{ji}\left|+\right|{\bar{\tilde{\alpha }}}_{ji}\left|\right)\right),$$

$\kappa^{*}>0$ is such that

$$\min_{1\leq i\leq n}\left(\frac{1}{{\bar{C}}_{i}{\bar{R}}_{i}}+{\bar{\tilde{a}}}_{i}\right)-\max_{1\leq i\leq n}\left(L_{i}\sum_{j=1}^{n}\left(\right|{\bar{\alpha }}_{ji}\left|+\right|{\bar{\tilde{\alpha }}}_{ji}\left|\right)\right)\geq \kappa^{*}>0,$$

*and*

$$\int_{t_{0}}^{\infty }\frac{W^{q}\left(t-t_{k},s-t_{k}\right)}{{\left(s-t_{0}\right)}^{1-q}}\sum_{i=1}^{n}\left(|\gamma_{i}\left(s\right)|+|{\tilde{\gamma }}_{i}\left(s\right)|\right)ds$$

$$+\sum_{j=1}^{k}\;\prod_{l=k-j+1}^{k}E_{q}\left(-\kappa^{*},t_{l}-t_{l-1}\right)$$

$$\int_{t_{k-j}}^{t_{k-j+1}}\frac{W^{q}\left(t-t_{k},s-t_{k-j}\right)}{{\left(s-t_{k_{j}}\right)}^{1-q}}\sum_{i=1}^{n}\left(|\gamma_{i}\left(s\right)|+|{\tilde{\gamma }}_{i}\left(s\right)|\right)ds<\infty .$$

*3. The unknown constants ${\tilde{P}}_{ik}$ are bounded such that $0<{\tilde{P}}_{ik}<1-\gamma_{ik},\;i=1,2,\ldots ,n,\;k=1,2,\ldots .$*

*Then the system (1) is (λ,A)- practically robustly exponentially stable with respect to the function h.*

**Example** **3.** *Consider the following 2-D uncertain impulsive fractional-like Hopfield neural network model*

(14) $$\left\{\begin{array}{c} D_{t_{k}}^{q}x_{i}\left(t\right)=-\left(\frac{1}{C_{i}\left(t\right)R_{i}\left(t\right)}+{\tilde{a}}_{i}\left(t\right)\right)x_{i}\left(t\right)\\ \;\;+\sum_{j=1}^{2}\left(\alpha_{ij}\left(t\right)+{\tilde{\alpha }}_{ij}\left(t\right)\right)f_{j}\left(x_{j}\left(t\right)\right)+\gamma_{i}\left(t\right)+{\tilde{\gamma }}_{i}\left(t\right),\;t\neq t_{k},\;k=0,1,\ldots ,\\ \Delta x\left(t_{k}\right)=\left(\begin{array}{cc} -\frac{3}{4}+{\tilde{P}}_{1k} & 0\\ 0 & -\frac{2}{3}+{\tilde{P}}_{2k} \end{array}\right)x\left(t_{k}\right),\;k=1,2,\ldots , \end{array}\right.$$

*where i=1,2, $t_{0}=0$, for which system (9) is the nominal system, and ${\tilde{a}}_{i}\in C^{q}\left[R_{+},\left(0,\infty \right)\right]$, ${\tilde{\alpha }}_{ij},{\tilde{\gamma }}_{i}\in C^{q}\left[R_{+},R\right]$, i,j=1,2,k=1,2,… and constants ${\tilde{P}}_{ik},\;i=1,2,\;k=1,2,\ldots$ are the uncertain parameters.*

*Then we have that, if all uncertain terms are bounded, and satisfied all conditions of Theorem 3, the system (9) is (λ,A)- globally practically robustly exponentially stable with respect to the function $h\left(x_{1},x_{2}\right)=|x_{1}\left|+\right|x_{2}|$.*

**Figure 3:**
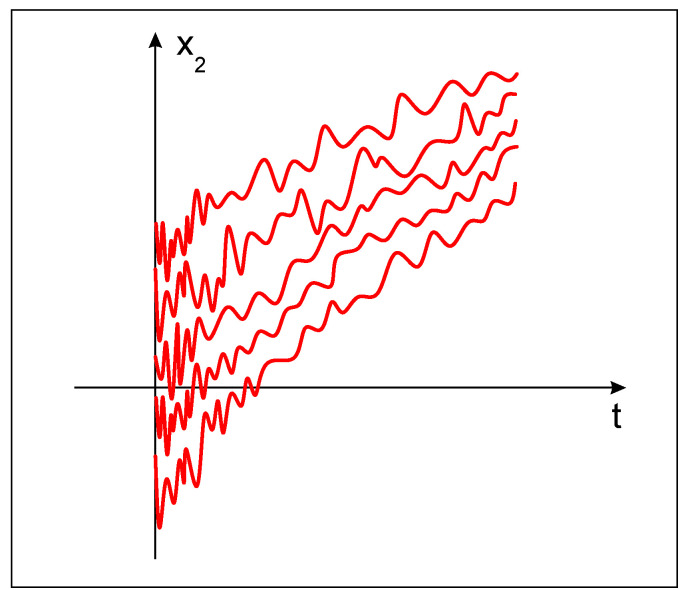
The unstable behavior of the state variable $x_{2}\left(t\right)$ of (14) for ${\tilde{P}}_{2k}=2,\;k=1,2,\ldots$ and λ=5, A=9.

*Note that, if some of the uncertain terms is unbounded, Theorem 3 cannot guarantee the robust practical stability of the fractional-like model (9). For example, for ${\tilde{P}}_{2k}=2,\;k=1,2,\ldots$, the unstable behavior of the model (14) is shown in Figure 3 for λ=5, A=9.*

## 6. Conclusions

In this paper a new class of impulsive neural network systems with FLDs has been proposed. Practical stability analysis is performed and efficient sufficient conditions are established. With this research we extend the results on impulsive neural network Hopfield-type models to the fractional-like case. In addition, the obtained results are applied to neural networks with uncertain valued of parameters. Since the use of FLDs overcome some difficulties in evaluating fractional derivatives the obtained results are more appropriate for applications.
